# Long non‐coding RNA H19 is responsible for the progression of lung adenocarcinoma by mediating methylation‐dependent repression of CDH1 promoter

**DOI:** 10.1111/jcmm.14533

**Published:** 2019-07-17

**Authors:** Li‐Ming Gao, Shu‐Feng Xu, Yue Zheng, Ping Wang, Ling Zhang, Shan‐Shan Shi, Tong Wu, Yang Li, Jing Zhao, Qi Tian, Xiao‐Bo Yin, Lei Zheng

**Affiliations:** ^1^ Department of Oncology the First Hospital of Qinhuangdao Qinhuangdao China; ^2^ Department of Respiratory the First Hospital of Qinhuangdao Qinhuangdao China; ^3^ Department of Pulmonary Chinese PLA General Hospital Beijing China; ^4^ Department of Respiratory Hebei Chest Hospital Shijiazhuang China; ^5^ School of Medicine Hebei Medical University Shijiazhuang China

**Keywords:** apoptosis, CDH1, epithelial‐mesenchymal transition, long non‐coding RNA H19, lung adenocarcinoma, methylation, proliferation

## Abstract

Lung adenocarcinoma is a common histologic type of lung cancer with a high death rate globally. Increasing evidence shows that long non‐coding RNA H19 (lncRNA H19) and CDH1 methylation are involved in multiple tumours. Here, we tried to investigate whether lncRNA H19 or CDH1 methylation could affect the development of lung adenocarcinoma. First, lung adenocarcinoma tissues were collected to detect CDH1 methylation. Then, the regulatory mechanisms of lncRNA H19 were detected mainly in concert with the treatment of overexpression of lncRNA H19, siRNA against lncRNA H19, overexpression of CDH1 and demethylating agent A‐5az in lung adenocarcinoma A549 cell. The expression of lncRNA H19 and epithelial‐mesenchymal transition (EMT)‐related factors as well as cell proliferation, sphere‐forming ability, apoptosis, migration and invasion were detected. Finally, we observed xenograft tumour in nude mice so as to ascertain tumorigenicity of lung adenocarcinoma cells. LncRNA H19 and methylation of CDH1 were highly expressed in lung adenocarcinoma tissues. A549 cells with silencing of lncRNA H19, overexpression of CDH1 or reduced CDH1 methylation by demethylating agent 5‐Az had suppressed cell proliferation, sphere‐forming ability, apoptosis, migration and invasion, in addition to inhibited EMT process. Silencing lncRNA H19 could reduce methylation level of CDH1. In vivo, A549 cells with silencing lncRNA H19, overexpression of CDH1 or reduced CDH1 methylation exhibited low tumorigenicity, reflected by the smaller tumour size and lighter tumour weight. Taken together, this study demonstrates that silencing of lncRNA H19 inhibits EMT and proliferation while promoting apoptosis of lung adenocarcinoma cells by inhibiting methylation of CDH1 promoter.

## 1 INTRODUCTION

Lung adenocarcinoma, a frequently occurring subtype of lung cancer, contributes to more than one million deaths on an annual basis globally.^1^ Lung adenocarcinoma is characterized by a heterogeneous group of tumours stemming from the smaller airways and occurring in peripheral lung tissues.^2^, ^3^ Metastasis, considered as a leading lethal cause of lung adenocarcinoma, can occur, only within months of diagnosis, in diverse organs in a swift manner.^4^, ^5^ Major therapies for lung adenocarcinoma conclude the use of erlotinib and gefitinib.^6^ However, unfortunately, lung adenocarcinoma is highly refractory to conventional radio‐ and chemotherapies, which poses a challenge to the treatment efficacy.^7^ Emerging evidence has reported the implication of long‐chain non‐coding RNAs (lncRNAs) in the development as well as progression of lung cancer.^8^

LncRNA H19, being an affluent and conserved transcript in the development of mammalian, is found in embryonic as well as extra‐embryonic cell lineages.^9^ Highly expressed lncRNA H19 was found to be responsible for metastasis of lung adenocarcinoma and to exhibit a negative correlation with the prognosis of patients with lung adenocarcinoma.^10^ Moreover, a recent study has demonstrated that lncRNA H19 promotes epithelial‐mesenchymal transition (EMT), a crucial process of cancer metastasis, by targeting miR‐484 in human lung cancer cells.^11^, ^12^ CDH1, a cell adhesion molecule also called E‐cadherin, plays a critical part in epithelial phenotype maintenance and functions as a gene that can suppress invasion.^13^ DNA methylation is considered to be a common event in lung cancer.^14^ As previously reported, silencing of CDH1 gene caused by hypermethylation of its promoter aided in the occurrence of lung primary adenocarcinoma.^15^ Intriguingly, lncRNA H19 was reported to inhibit the expression of E‐cadherin in bladder cancer and tongue squamous cell carcinoma.^16^, ^17^ From all the records mentioned above, it has been suggested that lncRNA H19 and CDH1 might be associated with the progression of lung adenocarcinoma. We downloaded lncRNA H19 expression in lung adenocarcinoma from the Cancer Genome Atlas (TCGA) database and found that compared with normal control tissues, lncRNA H19 was highly expressed in tissues of lung adenocarcinoma. Herein, we conducted this study to explore the effects of lncRNA H19 and CDH1 on lung adenocarcinoma with the involvement of methylation of CDH1, with the hope to raise the life quality of patients with lung adenocarcinoma.

## 2 MATERIALS AND METHODS

### 2.1 Ethics statement

This research study was conducted with the approval of the ethics committee of the First Hospital of Qinhuangdao. All patients participating in the study were provided with informed consent documentation, which they all subsequently signed. The animal experiment procedures were performed in accordance with strict protocols approved by the Institutional Animal Care and Use Committee.

### 2.2 Study patients

This study included 60 patients (aged 22‐67 years with a mean age of 45.18 ± 7.17 years; weighing 44‐84 kg, with a mean weight of 62.09 ± 7.58 kg; course of disease: 3‐60 months, with a mean course of 29.53 ± 9.86 months) pathologically diagnosed (cytology or histology) with primary lung cancer in the Department of Thoracic Surgery of the First Hospital of Qinhuangdao between 2014 and 2017. Lung adenocarcinoma tissues and adjacent tissues were collected from the patients (with each specimen weighing no <0.2 g) during lung resection. The patients were all new cases and included if: (a) patients pathologically diagnosed with lung adenocarcinoma; (b) patients who had undergone no cancer‐related treatment, such as radiotherapy and chemotherapy. The cancer tissues and adjacent tissues obtained through surgical resection were immediately placed into a liquid nitrogen tank.

### 2.3 Vector construction

siRNA single‐stranded oligonucleotide fragments (Sequences: siRNA‐1:5′‐TGACGGCGAGGACAGAGGAG‐3′, 5′‐CCCAGAGGGCAGCCATAGTG‐3′; siRNA‐2:5′‐CCCACAACAUGAAAGAAACTT‐3′, 5′‐AUUUCUUUCAUGUUGUGGGrl‐3′; siRNA‐3:5′‐GCUAGAGGAACCAGACCUUTT‐3′, 5′‐AAGGUCUGGUUCCUCUAGCTT‐3′) and negative control (NC) si‐NC sequence (5′‐ACAGAGCCTCGCCTTTGCCGAT‐3′, 5′‐CTTGCACATGCCGGAGCCGTT‐3′) were designed and synthesized based on human nucleotide lncRNA H19 sequences using shRNA online design system http://rnaidesigner. invitrogen. com/rnaiexpress/ (Invitrogen). The siRNA fragments were then dissolved by ddH_2_O into 100 μmol/L. After that, pairwise complementary single strands (5 μL for each) were selected, mixed and annealed. Next, four siRNA fragment mixtures were heated at 95°C for 5 minutes and then cooled at room temperature for 20 minutes to form double‐stranded fragments. The annealed double‐stranded siRNA was further diluted into 10 μmol/L concentration, after which the double‐stranded siRNA fragments were inserted into the siRNA expression vector pGFP‐V‐RS using a vector construction kit for connection at room temperature for 30 minutes, and the siRNA expression plasmid was constructed. Finally, the siRNA expression plasmid was converted to the competent cell DH5a, and three clones were selected in each conversion plate, respectively, for gene sequencing in order to verify whether the inserted fragment sequences in the recombinant clones were consistent with the designed siRNA oligonucleotide sequences. CDH1 overexpression vector was constructed as follows: Complete sequence analysis of the coding region of CDH1 was performed to detect whether there was a particularly complicated secondary structure and repetitive sequences in the CDH1 gene. Subsequently, based on the analysis results of gene sequencing, CDH1 single‐stranded oligonucleotide fragments were designed and synthesized, and restriction enzyme sites were added to the ends of the CDH1 sequence. PCR was used to splice the synthesized single‐stranded oligonucleotide fragments into a complete gene, after which the synthesized sequences were loaded into the pGFP‐V‐RS vector and converted into the competent cell DH5a. Next, gene sequencing was performed so as to verify whether the inserted fragment sequences in the recombinant clones were consistent with the requirements. The mutation sites in the gene sequences were repaired by overlapping PCR. Full‐length fragments after repair then underwent enzyme digestion by Xho I and EcoR and were connected to the target vector and converted into competent cell DH5a. Finally, sequence information of the target gene fragments in the recombinant clones was verified using sequencing to obtain correct CDH1 overexpression vector.

The siRNA interference vector and CDH1 overexpression vector were transiently cotransfected: Cells were seeded into each well of the 6‐well plate (5 × 10^5^ cells/well), followed by culturing overnight, with the cell growth observed. When the cell confluence reached approximately 80%, cell transfection was conducted. Serum‐free Opti‐MEM was used to wash the cells twice, and 1.5 mL Opti‐MEM was added. Next, 250 μL Opti‐MEM was used to dilute 3 μg interference plasmid and NC plasmid, respectively, followed by slight mixing and addition of 1 μg overexpression vector. A total of 10 μL Lipofectamine 2000 reagent was diluted by 250 μL Opti‐MEM, mixed gently and incubated at room temperature for 5 minutes. The diluted plasmids and Lipofectamine 2000 were gently mixed and allowed to stand at room temperature for 20 minutes. Afterwards, plasmid mixture (500 μL each tube) was slowly added to each cell well and gently mixed. Following 6 hours of culturing in an incubator of 37°C with 5% CO_2_, the previous medium was replaced by complete culture solution (without antibiotics) and further cultured in a 37°C incubator with 5% CO_2_ overnight. At the 3rd day, the expression of enhanced green fluorescent protein (EGFP) protein was observed at 488 nm under a fluorescence microscope, and the expressions of lncRNA H19 and CDH1 in the cells were determined by reverse‐transcription quantitative polymerase chain reaction (RT‐qPCR) in order to detect the interference efficiency and overexpression efficiency.

### 2.4 Methylation‐specific polymerase chain reaction (MSP) assay

Lung adenocarcinoma cancer tissues and adjacent tissues (each n = 60) were collected. Initially, the tissue DNA was extracted by conventional phenol‐chloroform‐isoamyl alcohol method and the concentration and purity of DNA were determined using ultraviolet spectrophotometry. Next, DNA modification and purification were performed with hydroquinone and sodium bisulfite (Sigma‐Aldrich, SF) and DNA Clean‐up System (Promega). Methylation‐specific PCR reaction was as follows: The reaction system was 25 μL, including 2 μL sample DNA, 2.5 μL 10 × PCR buffer, 0.5 μL forward primer and 0.5 μL reverse primer, 2 μL dNTP, 0.2 μL Taq enzyme and 17.3 μL double‐distilled water. Reaction conditions were as follows: pre‐denaturation at 95°C for 5 minutes, and 35 cycles of denaturation at 94°C for 30 seconds, at 57°C for 45 seconds, at 72°C for 30 seconds and extension at 72°C for 10 minutes. The annealing temperature of methylation reaction was 57°C, and that of non‐methylation reaction was 54°C, with double‐distilled water used as a blank control and 1000 bp D L2000 as a molecular mass marker. Primer sequences were as follows: (a) methylation primers: forward primer: 5′‐TTAGGTTAGAGGGTTATCGCGT‐3; reverse primer: 5′‐TAACTAAAAATTCACCTACCGAC‐3; amplification product was 115 bp; (b) non‐methylation primers: forward primer: 5′‐TAATTTTAGGTTAGAGGGTTATTGT‐3′; reverse primer: 5 ′‐CACAACCAATCAACAACACA‐3′; amplification product was 97 bp. The obtained PCR product was 5 μL, which then underwent 2% agarose gel electrophoresis at 100 V for 40 minutes. Finally, the electrophoresis results were analysed by a laser scanner (Pharmasia Company). The experiment was repeated three times (also applicable to cell experiments).

### 2.5 Immunohistochemistry

The specimens were fixed with formaldehyde for more than 24 hours, conventionally dehydrated by gradient alcohol (70%, 80%, 90%, 95%, 100%; 1 minute each), cleared by xylene twice, 5 minutes each, immersed and embedded in paraffin and sliced into 4‐μm sections. The sections were baked in a 60°C oven overnight, deparaffinized with xylene, and immersed in gradient alcohol (100%, 95%, 80%, 70%; 5 minutes each) and running water for 5 minutes and washed with phosphate‐buffered saline (PBS) three times, 3 minutes each. Next, the sections were heated with 0.01 mol/L citric acid buffer for antigen retrieval (10 minutes) and cooled down to room temperature, followed by 3 PBS washes, 3 minutes each. Endogenous peroxidase was blocked by immersing the sections in 0.3% H_2_O_2_‐formaldehyde solution for 20 minutes. Following three times of PBS washes, the sections were blocked with 10% goat serum (36119ES03, Yeasen Company) at room temperature for 10 minutes. CDH1 antibody (1:50; ab1416, Abcam Inc) working solution was added to the sections, followed by incubation overnight at 4°C, with the primary antibody replaced by PBS as NC. Horseradish peroxidase (HRP)‐labelled goat anti‐rabbit secondary antibody immunoglobulin G (IgG; 1:1000; ab6721, Abcam Inc) was dropped to the sections and incubated at room temperature for 30 minutes. Next, 3,3′‐diaminobenzidine (DAB, Beyotime Biotechnology Co.) was added for coloration for 5 minutes, which was observed under a microscope. After that, the sections were rinsed with running water for 5 minutes, counterstained with haematoxylin for 3 minutes, differentiated by 1% ethanol‐hydrochloric acid for 5 seconds, rinsed in running water for 10 minutes to return blue colour, mounted by neutral gum, observed under an optical microscope and photographed. The cells with brown‐yellow cytoplasm or membrane were considered to be positive. Five fields of vision were randomly selected in each section to calculate the positive rate.

### 2.6 RT‐qPCR

The total RNA of cells was extracted using one‐step method with reference to Trizol kit (15596‐026, Invitrogen), of which the purity and optical density (OD) ratio at 260 nm and at 280 nm (OD_260_/ OD_280_) were determined in connection with a nucleic acid and protein analyzer (BioPhotometer D30, Eppendorf). The value of OD_260_/ OD_280_ ranging from 1.8 to 2.0 was indicative of a high purity. Based on the instructions of reverse transcription kit (k1622, Fermentas), the obtained RNA was reversely transcribed into complementary DNA (cDNA). The reaction conditions consisted of: at 70°C for 5 minutes, ice bath for 3 minutes, at 37°C for 60 minutes, and at 95°C for 10 minutes. The obtained cDNA was stored in a −20°C refrigerator. The sequences of lncRNA H19, EMT‐related factors CDH1, N‐cadherin, vimentin, proliferating cell nuclear antigen (PCNA), Ki67 and apoptosis‐related genes B‐cell lymphoma/lewkmia‐2 (Bcl‐2), Bax (Bcl‐2‐associated X protein) and cleaved caspase‐3 (Table 1) were synthesized by Shanghai Genechem Co., Ltd. A fluorescence qPCR kit (Takara Biotechnology Ltd.) was used to determine mRNA expression of each gene, with the reaction system as follows: 5.3 μL 2 × Taq MasterMix, 1 μL forward primer (5 µmol/L), 1 μL reverse primer (5 µmol/L), 1 μL cDNA and 11.7 μL RNase‐free H_2_O. The reaction conditions concluded: pre‐denaturation at 95°C for 5 minutes, 35 cycles of denaturation at 94°C for 45 seconds, annealing at 56°C for 45 seconds and extension at 72°C for 45 seconds. A fluorescence qPCR instrument (ABI7500, ABI Company) was employed for gene determination. With the glyceraldehyde‐3‐phosphate dehydrogenase (GAPDH) gene used as the internal reference, 2^−ΔΔCt^ method was used to calculate relative expressions of the target genes with the following formula: ΔΔCT = (mean Ct value of the target gene in the experiment group−mean Ct value of housekeeping gene in the experiment group)−(mean Ct value of the target gene in the control group−mean Ct value of housekeeping gene in the control group). The experiment was repeated three times independently. The aforementioned method was equally applicable to the mRNA determination among the cells.

**Table 1:** Primer sequences for RT‐qPCR

Gene	Sequences
lncRNA H19	F: 5′‐ATCGGTGCCTCAGCGTTCGG‐3′
	R: 5′‐CTGTCCTCGCCGTCACACCG‐3′
CDH1	F: 5′‐ACACCATCCTCAGCCAAGA‐3′
	R: 5′‐CGTAGGGAAACTCTCTCGGT‐3′
N‐cadherin	F: 5′‐CAACTTGCCAGAAAACTCCAGG‐3′
	R: 5′‐ATGAAACCGGGCTATCTGCTC‐3′
vimentin	F: 5′‐CGCCAGATGCGTGAAATGG‐3′
	R: 5′‐ACCAGAGGGAGTGAATCCAGA‐3′
PCNA	F: 5′‐GCGCAGAGGGTTGGTAGTTG‐3′
	R: 5′‐CCCGATTCACGATGCAGAA‐3′
Ki67	F: 5′‐AGGACTTTGTGCTCTGTAACC‐3′
	R: 5′‐CTCTTTTGGCTTCCATTTCTTC‐3′
Bcl‐2	F: 5′‐CACGCTGGGAGAACA‐3′
	R: 5′‐CTGGGAGGAGAAGATG‐3′
Bax	F: 5′‐GGATGCGTCCACCAAGAAG‐3′
	R: 5′‐GCCTTGAGCACCAGTTTGC‐3′
caspase‐3	F: 5′‐ATAACCTTTTAGGCTGGTGG‐3′
	R: 5′‐AGAGCAGAAAGA GGTGAGAGA‐3′
GAPDH	F: 5′‐ACAACAGCCTCAAGATCATCAG‐3′
	R: 5′‐GGTCCACCACTGACACGTTG‐3′

Abbreviations: Bax, Bcl‐2‐associated X protein; Bcl‐2, B‐ cell lymphoma/lewkmia‐2; CDH1, E‐cadherin; F, forward; GAPDH, glyceraldehyde‐3‐phosphate dehydrogenase; lncRNA H19, long non‐coding RNA H19; PCNA, proliferating cell nuclear antigen; R, reverse; RT‐qPCR, reverse‐ transcription ‐quantitative polymerase chain reaction.

### 2.7 Western blot analysis

The obtained tissue specimens were ground into homogenate at 1610 × g with protein lysate (R0010, Beijing Solarbio Science & Technology Co., Ltd. The proteins were ice‐bathed for 30 minutes at 4°C and centrifuged at 25 764 *g* for 15 minutes with the supernatant collected. The concentration of the proteins was determined using the bicinchoninic acid (BCA) kit (23225, Pierce) and then adjusted to 1 μg/μL. The treated proteins were added to the sample loading wells, with 20 μg per well. Next, 10% sodium dodecyl sulfate polyacrylamide gel electrophoresis (SDS‐PAGE; Beijing Solarbio Science & Technology Co., Ltd.) was performed to separate the proteins. The electrophoresis started at 60 V, and the voltage was changed to 100 V after the proteins entered the separation gel. When the samples approached the bottom of the separation gel, the electrophoresis was terminated. The proteins on the gel were then transferred to polyvinylidene fluoride (PVDF) membranes (HVLP04700, Merck Millipore) using semi‐dry electrophoretic transfer. Ponceau (P0012, Beijing Solarbio Science & Technology Co., Ltd.) staining was performed with protein transfer observed. Afterwards, the membranes were washed twice with Tris‐buffered saline Tween‐20 (TBST) and blocked with 5% skim milk for 2 hours, followed by 3 TBST washes. Primary antibodies CDH1 (1:50; ab1416), vimentin (1:1000; ab92547), N‐cadherin (1:1000; ab6528), Bcl‐2 (1:1000; ab32124), Bax (1:10000; ab32503), cleaved caspase‐3 (1:500, ab13847), PCNA (1:1000, ab92553) and GAPDH (1:1000; ab8245), all purchased from Abcam Inc, were then added to the membranes, followed by incubation in a 37°C refrigerator overnight. Following 3 TBST rinses (10 minutes each), the membranes were added with HRP‐labelled secondary antibody mouse anti‐human IgG (1:2000; ab6721, Abcam Inc). After 2 hours of incubation at room temperature, the membranes were washed with TBST three times, 10 minutes each time, followed by development with DAB and photographing using a gel imager (Gel Doc XR, Bio‐Rad, Inc). The ratio of the grey value of the target band to the internal reference (GADPH) band was used as the relative expression of the protein. This method was equally applicable to the protein expression determination and cell experiments.

### 2.8 Cell treatment

Normal lung cell line HFL1 and lung adenocarcinoma cell lines A549, H1299, PC9, PG49 and NCl‐H1975 (purchased from the Chinese Academy of Sciences) were routinely cultured, detached and centrifuged, suspended and seeded in a 6‐well plate. Upon cell confluence of 80%, DNAs were extracted from all the above lung adenocarcinoma cell lines. The previously designed PCR primers for CDH1 methylation and non‐methylation were used to determine DNA methylation level of CDH1 using MSP assay. Next, part of the cells was gently scraped out with a 200‐μL pipette and rinsed three times with PBS. The medium containing 10 μmol/L 5‐Aza was added to the cells, which were then cultured in a 37°C thermostat with 5% CO_2_. Following 48 hours of culturing, RT‐qPCR and Western blot analysis were used to determine mRNA and protein expression of CDH1 in the same way with the aforementioned method. After screening, cell suspension of the most suitable cell line (A549) was transferred to Dulbecco's modified Eagle's medium (DMEM) containing 20% foetal bovine serum (FBS) for further culturing in an incubator with 5% CO_2_ at 37°C, with medium changed once every 2 or 3 days.

A549 cells of the third generation were assigned into seven groups: mock group (without any treatment), oe‐NC group (cells transfected with 0.4 pmol/μL blank vector), oe‐CDH1 group (cells transfected with 0.4 pmol/μL CDH1 overexpression plasmid), dimethylsulfoxide (DMSO) group (cells cultured with DMSO), 5‐Aza group (cells cultured with 10 pmol/μL 5‐Aza, a demethylating agent), si‐NC group (cells transfected with 0.4 pmol/μL si‐NC plasmid) and si‐lncRNA H19 group (cells transfected with 0.4 pmol/μL si‐lncRNA H19 plasmid). The cells in logarithmic growth were seeded into a 6‐well plate, and when the cell density reached 30%‐50%, the cells were transfected according to the instructions of Lipofectamine 2000 (Invitrogen). A total of 250 μL serum‐free medium Opti‐MEM (Gibco) was used to dilute 100 pmol oe‐CDH1, si‐lncRNA and NC (the final concentration was 50 nmol/L), followed by sufficient mixing and incubation at room temperature for 5 minutes. A total of 5 μL Lipofectamine 2000 was diluted by 250 μL serum‐free medium, mixed and incubated at room temperature for 5 minutes. The above two mixtures were mixed, incubated at room temperature for 20 minutes and added to the cell culture plate. After incubation at a 37°C incubator with 5% CO_2_ for 6‐8 hours, the complete medium was used to replace the previous medium for 24‐48 hours of further culturing for subsequent experimentation purpose.

### 2.9 Cell counting kit (CCK)‐8 assay

Cell proliferation was detected using CCK‐8 (GM‐040101‐5, Dojindo Laboratories) based on its protocol. Cells in logarithmic growth after transfection were collected, rinsed with PBS, treated with trypsin, washed with additional PBS and suspended. After the cell concentration was adjusted to 5 × 10^3^ cells/μL, the cells were transferred to a 96‐well plate, with six duplicated wells set for each group. The plate was then placed in a 37°C incubator with 5% CO_2_ in which the cells were cultured for consecutive 2 days. At the last 4 hours, 10 μL CCK‐8 solution was added to each well for further culturing, after which spectrophotometer (UV‐1800A, Macylab) was employed to measure OD value at the wavelength of 450 nm based on the following formula: OD = OD _experiment well_‐OD _bank well_. The experiment was repeated three times.

### 2.10 Sphere‐forming assay

Human lung adenocarcinoma A549 cell line in each group underwent sphere‐forming culture. With the addition of high glucose DMEM and 10% FBS, the A549 cells after amplification transfection were cultured in vitro in a 37°C thermostat with 5% CO_2_. When the cell confluence reached 80%, 0.25% trypsin was used to treat the cells, which were then collected, suspended and counted. Next, the cells were seeded in a 6‐well ultra‐low attachment plate and cultured in a 37°C incubator with 5% CO_2_. After 6 days of culturing, the tumour spheres grew into 50 μm and the cell suspension was collected. Following low‐speed centrifugation at 64 *g* for 5 minutes, the tumour spheres precipitated at the bottom of the centrifuge tube, while the single cells with lighter weight were still suspended in the supernatant. After the upper cell suspension was removed, PBS was slightly added to resuspend the precipitates. Pure glomus cells were obtained by two repetitions of the above steps, treated with 0.25% trypsin/0.02% ethylenediamine tetraacetic acid (EDTA), incubated at a 37°C incubator for 2 minutes and resuspended. Finally, the cells were counted and the average value was taken from three independently repeated experiments.

### 2.11 Flow cytometry

Annexin V‐fluorescein isothiocyanate (FITC)/propidium iodide (PI) double staining was used to detect cell apoptosis. After 48 hours of transfection, cells were collected and treated with 0.25% trypsin. Following 48‐hours culture in a 37°C incubator with 5% CO_2_, the cells were harvested, rinsed with PBS twice, centrifuged and resuspended in 200 μL binding buffer. A total of 10 μL Annexin V‐FITC and 5 μL PI were gently mixed, followed by reaction in the dark for 15 minutes and addition of 300 μL binding buffer. A flow cytometry (6HT, Cellwar Bio‐technology) was employed to detect cell apoptosis at the excitation wavelength of 488 nm.

### 2.12 Scratch test

Cells in logarithmic growth were collected after 48 hours of transfection and seeded in a 6‐well plate at a density of 1 × 10^6^/well. The plate was then transferred to a 37°C incubator with 5% CO_2_. Upon cell confluence of approximately 95%, a 20‐μL micropipette tip was used to make vertical line scratches in the middle of culture wells. D‐Hank's solution was used to wash off the falling cells, following which the cells were further cultured with serum‐free medium. At 0 and 36 hours after scratching, the samples were collected. Three fields of vision (100×) were randomly selected under a phase‐contrast microscope and photographed to compare scratch healing among each group. The number of migrating cells was calculated, with the average value of the three independent experiments taken as the final migrating cell number.

### 2.13 Transwell assay

After 48 hours of transfection, cells were starved in serum‐free medium for 12 hours, detached, rinsed with PBS twice and suspended with serum‐free medium Opti‐MEMI (31985‐070, Invitrogen) containing 10 g/L bovine serum albumin (BSA), with the cell concentration adjusted to 2 × 10^6^ cells/mL. A 24‐well plate with 8‐μm Transwell chambers (3413, Corning Glass Works, Corning) was prepared, with three chambers set for each group. Prior to the experiment, 50 μL Matrigel (40111ES08, Sigma‐Aldrich, SF) was used to cover the chambers, 48 hours after which 200 μL single cell suspension (4 × 10^4^ cells) in each group was added to the apical chambers and 650 μL G‐DMEM culture medium containing 10% FBS was added to the basolateral chambers. Following 12 hours of culturing in a 37°C incubator with 5% CO_2_, the chambers were taken out, rinsed with PBS, immersed in formaldehyde solution, fixed at room temperature for 30 minutes and then stained with 0.1% crystal violet for 20 minutes. Afterwards, cells on the upper layer of the microporous membrane were gently removed with cotton swabs. The cells were observed under an inverted optical microscope with images captured. Four fields of vision were randomly selected in order to count the cells passing through the microporous membrane, with the average value calculated. The experiment was repeated three times.

### 2.14 Immunofluorescence assay

At one week after plasmid in each group was transfected into A549 cells, cell slides were prepared and fixed for 20 minutes in mixed cold fixative freshly prepared with methanol and acetone (volume ratio of 3:1). After two times of washes with distilled water, the cells were blocked with H_2_O_2_ for 30 minutes, followed by 3 PBS washes, 5 minutes each. Next, primary antibodies CDH1 (1:50, ab1416, Abcam Inc) and N‐cadherin (1:1000, ab6528, Abcam Inc) were added to the cells, followed by incubation at 4°C overnight and 3 PBS washes, 5 minutes each. Subsequently, FITC‐labelled goat anti‐rabbit IgG used as secondary antibody was added to the cells, incubated at 37°C for 30 minutes, washed three times with PBS and observed under a fluorescence microscope. PBS was used as NC instead of primary antibodies.

### 2.15 RNA fluorescence in situ hybridization (RNA‐FISH)

FISH technique was used to identify the subcellular localization of lncRNA H19 in lung adenocarcinoma cells in accordance with the instructions of Ribo^TM^ lncRNA FISH Probe Mix (Red) (Ribobio). A cover glass was placed into a 24‐well plate, and cells were seeded to the plate at a density of 6 × 10^4^/well to allow the cell confluence to reach approximately 80%, after which the cover glass was taken out, rinsed with PBS and fixed with 1 mL 4% polyformaldehyde at room temperature. Following treatment with protease K (2 μg/mL), glycine and phthalide reagent, 250 μL prehybridization solution was added to the cells for incubation for a period of 1 hours at 42°C. Next, the prehybridization solution was replaced with 250 μL hybridization solution containing probes (300 ng/mL), followed by hybridization at 42°C overnight. After 3 PBST rinses, 4',6‐diamidino‐2‐phenylindole (DAPI) dye liquor diluted by PBST (1:800) was added to the 24‐well plate to stain the nucleus for 5 minutes. Following 3 PBST washes, 3 min each, fluorescence quenching agent was used to seal the cover glass, which was then observed and photographed under a fluorescence microscope (Olympus).

### 2.16 Chromatin immunoprecipitation (ChIP)

A ChIP kit (Merck Millipore) was used to study the enrichment of DNA methyltransferase 1 (DNMT1) and DNA methyltransferase 3A (DNMT3A) in the promoter region of CDH1 gene. A549 cells were collected, fixed in 1% formaldehyde at room temperature for 10 minutes, randomly cracked through ultrasonic treatment after 15 cycles of cross‐linking (10 seconds each ultrasonic, with an interval of 10 seconds) and centrifuged at 30237 *g* at 4°C. The supernatant was collected and separately placed into three tubes, followed by respective addition of NC antibody, namely, IgG of normal mice and specific rabbit antibodies of target protein, DNMT1 (1:100; ab13537, Abcam Inc) and DNMT3A (1:100; ab2850, Abcam Inc) and incubation at 4°C overnight. Following a short centrifugation, the supernatant was discarded and the non‐specific complex was washed, followed by cross‐linking at 65°C overnight. DNA fragments were retrieved using phenol‐chloroform extraction and purification. Specific primers of the CDH1 promoter region (forward primer: 5′‐AGTCCCACAACAGCATAGGG‐3′; reverse primer: 5′‐TTCTGAACTCAGGCGATCCT‐3′) were used to test the binding of DNMT1 and DNMT3A to CDH1 promoter region.

### 2.17 Luciferase reporter gene assay

The relationship between lncRNA H19 and CDH1 was detected using luciferase reporter gene assay. Based on the binding sequence between the sequence of CDH1 promoter and lncRNA H19, the wild target sequence (CDH1 wt) and mutation sequence (CDH1 mut) were designed and chemosynthesized. The restriction sites Xho I and Not I were supplemented to the ends of the sequences, and the synthesized segments were cloned to the PUC57 vector. Following positive cloning and recombinant plasmid identification using DNA sequencing, the segments were subcloned to the psi‐CHECK‐2 vector and transferred to the coli DH5α cells, followed by plasmid amplification. The plasmids were then extracted according to a plasmid mini kit (Omega Bio‐tek Inc). Cells were seeded into a 6‐well plate at a density of 2 × 10^5^/well. After adhering to the wall, the cells were transfected according to the following method. After successful transfection, the cells were cultured for 48 hours and collected. The changes in luciferase activity of CDH1 3'‐UTR in cells brought by lncRNA H19 were detected by the operation method provided by the dual luciferase detection kit of Genecopoeia. The fluorescence intensity was detected by a Glomax20/20 luminometer fluorescence detection instrument (Promega Corporation), and the expressions of normal CDH1 (N‐CDH1) and methylated CDH1 (M‐CDH1) were detected by MSP assay. The experiment was repeated three times.

### 2.18 Xenograft tumour in nude mice

Cells in each group were conventionally passaged for about one month after transfection. Lung adenocarcinoma cells were subcutaneously injected into nude mice. A total of 42 BLAB/CA nude mice (aged 4‐6 weeks and weighing 20‐24 g) purchased from Laboratory Animal Center of Beijing Medical University were randomly assigned into seven groups, namely, mock, oe‐NC, oe‐CDH1, DMSO, 5‐Aza, si‐NC and si‐lncRNA H19 groups. Mice in each group were injected with the A549 cells previously transfected with corresponding plasmids. Each nude mouse was subcutaneously injected with 100 μL cell suspension via the right axillary, with 1 × 10^7^ cells inoculated in each mouse. Tumour formation in nude mice was observed once every week: The tumour was weighed using an electronic scale, the volume of the tumour was measured using a vernier calliper, and changes of spirit, diet and activity of the mice were observed. At the 4th week after inoculation, mice were killed and the tumour was completely separated under aseptic conditions, photographed and weighed. The weight and volume of the transplanted tumours in nude mice (volume = 1/2 × width^2^) were measured, and the differences among each treatment group were compared. Tumour tissues were fixed with 10% formaldehyde and paraffin embedded. Haematoxylin‐eosin (HE) staining and immunohistochemistry were employed for detection of microvascular and lymphatic micro‐vessels. Some of the tumour tissues were frozen and preserved in liquid nitrogen for further use, and 4‐μm sections were used for immunohistochemistry. Cells with brown‐yellow cytoplasm or membrane were considered to be positive. Five fields of vision were randomly selected in each section, and lymphatic micro‐vessel density (LVD) was measured as follows: The distribution of the vascular or lymphatic vessels of the whole section was observed under low magnification (× 100), and then, three regions in which the brown‐yellow granules were the densest were selected in the tumour tissues, namely, ‘hot spots’. Next, lymphatic vessel endothelial hyaluronan receptor (LYVE)‐1 positive cells in each region were counted under high magnification (× 400), and the average value of the five regions was taken as the micro‐vessel density (MVD) or LVD of the tumour section. Any brown blood vessel, lymphatic endothelial cells or endothelial cell mass separated from adjacent micro‐vessels, tumour cells or other connective tissues was counted by independent micro‐vessels or lymphatic micro‐vessels. Vessels with a diameter of more than 8 red blood cells and with a distinct muscular layer were not considered as micro‐vessels; vessels composed of a single layer of endothelial cells, with no smooth muscle cells around and no red cells in the lumen, were regarded as lymphatic vessels. The histological types, the distribution of positive cells and immunohistochemical features were observed using double‐blind method by two pathologists.

### 2.19 Statistical analysis

The SPSS 21.0 software (IBM Corp., Armonk, NY, USA) was used for data analysis. Measurement data were presented as mean ± standard deviation. The *t* test was used for data comparison between two groups and one‐way analysis of variance (ANOVA) for comparisons among multiple groups. Enumeration data were demonstrated as percentage or ratio and compared by chi‐square analysis. *P* < .05 was considered to be statistically significant difference.

## 3 RESULTS

### 3.1 LncRNA H19 and methylation of CDH1 are highly expressed in lung adenocarcinoma tissues

**Figure 1:**
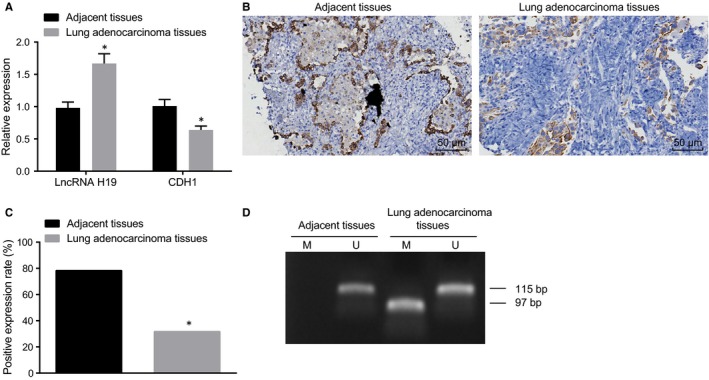
RT‐qPCR, immunohistochemistry and MSP show high expression of lncRNA H19, poor expression of CDH1 and high methylation of CDH1 in lung adenocarcinoma tissues. A, expression of lncRNA H19 and CDH1 in the lung adenocarcinoma tissues detected by RT‐qPCR; B, expression graph of CDH1 measured by immunochemistry (200×); C, statistical plot of CDH1 expression determined by immunohistochemistry; D, CDH1 methylation bands detected by MSP, M refers to amplified fragment of methylation of CDH1 (97 bp), and U refers to amplified fragment of non‐methylation of CDH1 (115 bp). RT‐qPCR, reverse‐transcription quantitative polymerase chain reaction; lncRNA H19, long non‐coding RNA H19; CDH1, E‐cadherin; MSP, methylation‐specific polymerase chain reaction. *, *P* < .05 *vs.* the adjacent tissues; the measurement data are presented as mean ± standard deviation, analysed by Student's *t* test. n = 60. The experiment was independently repeated three times

In order to investigate the correlation between CDH1, CDH1 methylation and lung adenocarcinoma, we initially performed RT‐qPCR to determine the expressions of cellular factors in the lung adenocarcinoma tissues and adjacent tissues. The results exhibited that compared with the adjacent tissues, the expression of lncRNA H19 was significantly elevated and the mRNA expression of CDH1 was notably decreased in the lung adenocarcinoma tissues (*P* < .05) (Figure 1A). Next, we used immunohistochemistry to determine the expression of CDH1 in the lung adenocarcinoma tissues and adjacent tissues. The results showed that among the 60 cancer tissues, 41 tissues had no CDH1 expression or cytoplasm/membrane staining, with negative expression found in immunohistochemistry. Among the 60 adjacent tissues, 13 cases had negative CDH1 expression and the remaining 47 cases showed positive CDH1 expression which was mainly located in cytoplasm/membrane, presenting in fine yellow granules (Figure 1B). Compared with the adjacent tissues, the rate of positive protein expression of CDH1 in the lung adenocarcinoma tissues was significantly lower (Figure 1C), which was consistent with the results shown in RT‐qPCR.

We determined methylation of CDH1 in 60 cases of lung adenocarcinoma tissues and 60 cases of adjacent tissues using MSP assay. No methylation of CDH1 was detected in all the 60 adjacent tissues, while among the 60 lung adenocarcinoma tissues, 17 cases showed methylation of CDH1 promoter, accounting for 28.33%. Compared with the adjacent tissues, the lung adenocarcinoma tissues exhibited a notable increase in methylation of CDH1 promoter (Figure 1D, Table 2). The aforementioned results suggest that methylation of CDH1 promoter is correlated with the occurrence of lung cancer.

**Table 2:** MSP assay shows that methylation of CDH1 promoter is highly expressed in lung adenocarcinoma tissues

Group	Positive case	Positive rate (%)
Adjacent tissues (n = 60)	0	0
Lung adenocarcinoma tissues (n = 60)	17^a^	28.33*

Abbreviations: CDH1, E‐cadherin; MSP, methylation‐specific polymerase chain reaction; n, number.

*P < 0.05 compared with the adjacent tissues.

### 3.2 A549 cell line is selected for cell transfection with silencing and overexpression plasmids of CDH1 successfully constructed

**Figure 2:**
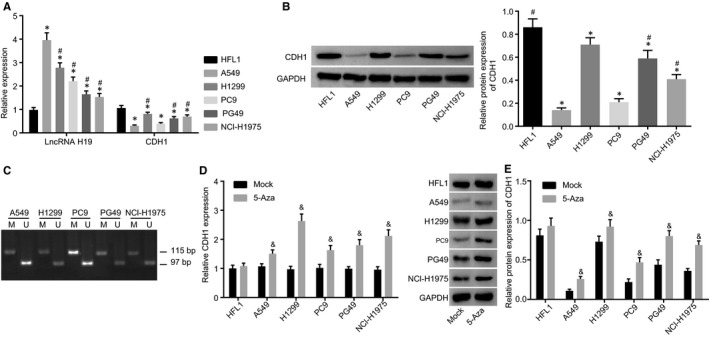
Determination by RT‐qPCR and Western blot analysis show that A549 cell line with the highest lncRNA H19 expression and the second lowest CDH1 expression was selected for cell transfection. A, expression of lncRNA H19 and CDH1 measured by RT‐qPCR; B, protein band and statistical chart of CDH1 expression determined by Western blot analysis; C, CDH1 methylation detected by MSP. M refers to amplified fragment of methylation of CDH1 (97 bp), and U refers to amplified fragment of non‐methylation of CDH1 (115 bp). D, the mRNA expression of CDH1 determined by RT‐qPCR; E, the protein expression of CDH1 detected by Western blot analysis. RT‐qPCR, reverse‐transcription quantitative polymerase chain reaction; lncRNA H19, long non‐coding RNA H19; CDH1, E‐cadherin; GAPDH, glyceraldehyde‐3‐phosphate dehydrogenase; MSP, methylation‐specific polymerase chain reaction. *P < .05 vs the HFL1 cell line; ^#^ *P* < .05 vs the A549 cell line; ^&^ *P* < .05 vs the mock group. The measurement data are presented as mean ± standard deviation, analysed by one‐way analysis of variance. n = 3. The experiment was independently repeated three times

We performed RT‐qPCR (Figure 2A) and Western blot analysis (Figure 2B) to determine the expressions of lncRNA H19 and CDH1 so as to select a cell line with the highest lncRNA H19 expression and lowest CDH1 expression for further experimentation. The results showed that compared with the normal lung cell line HFL1, among the lung adenocarcinoma cell lines, A549 cell line had the highest lncRNA H19 expression and the second lowest CDH1 expression (A549 cell line only slightly higher than PC9 cell line; *P* > .05). Therefore, A549 cell line was selected for further experimentation. Subsequently, we extracted DNA from each lung adenocarcinoma cell line to design PCR primers for methylation of CDH1. MSP assay was performed to determine the DNA methylation level of CDH1, exhibiting that each lung adenocarcinoma cell line had methylation of CDH1 promoter with varying degrees and that in comparison with the other lung adenocarcinoma cell lines, the A549 cell line showed a highest methylation level of CDH1 promoter (Figure 2C). We collected some cells from each cell line and cultured them in medium containing 10 μmol/L 5‐Aza. Next, RT‐qPCR (Figure 2D) and Western blot analysis (Figure 2E) were used to determine the mRNA and protein expression of CDH1 in the above cells treated with demethylating agent. Compared with the mock group, the mRNA and protein expressions of CDH1 in each cell line in the 5‐Aza group were significantly up‐regulated (*P* < .05).

**Figure 3:**
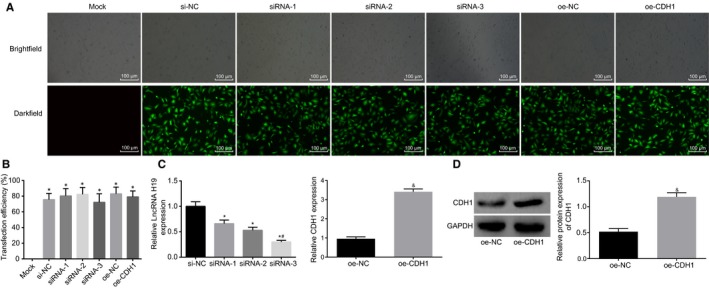
Silencing and overexpression plasmids of CDH1 are successfully constructed. A, transfection efficiency of cells in each group detected by immunofluorescence assay under a fluorescence microscope. B, statistical chart of transfection efficiency of cells in each group, *P* < .05 vs the mock group; C, expression of lncRNA H19 and CDH1 detected by RT‐qPCR after transfection, *P < .05 vs the si‐NC group; ^#^ *P* < .05 vs the siRNA‐1 group and the siRNA‐2 group; ^&^ *P* < .05 vs the oe‐NC group; D; the expression of CDH1 in the oe‐CDH1 group, ^&^ *P* < .05 vs the oe‐NC group. RT‐qPCR, reverse‐transcription quantitative polymerase chain reaction; lncRNA H19, long non‐coding RNA H19; CDH1, E‐cadherin; GAPDH, glyceraldehyde‐3‐phosphate dehydrogenase; NC, negative control. The measurement data are presented as mean ± standard deviation, analysed by one‐way ANOVA. n = 3. The experiment was independently repeated three times

In addition, we constructed the lncRNA H19 silencing plasmid containing green fluorescence gene, CDH1 overexpression plasmid and blank control plasmid. The morphology of cells in each group was clearly observed at 488 nm under a fluorescence microscope (Figure 3A). No fluorescence was detected in the mock group, while the si‐NC, siRNA‐1, siRNA‐2, siRNA‐3, oe‐NC and oe‐CDH1 groups exhibited a large amount of fluorescence. According to the statistical results, the transfection efficiency in the si‐NC, siRNA‐1, siRNA‐2, siRNA‐3, oe‐NC and oe‐CDH1 groups was over 70% (Figure 3B). Next, we further determined the expressions of lncRNA H19 and CDH1 using RT‐qPCR. In comparison with the si‐NC group, the siRNA‐1, siRNA‐2 and siRNA‐3 groups had significantly reduced lncRNA H19 expression and the lowest lncRNA H19 expression was found in the siRNA‐3 group among the four groups, with an inhibitory efficiency more than 70%. Therefore, siRNA‐3 was selected for further experimentation. The oe‐CDH1 group had a higher CDH1 mRNA expression than the oe‐NC group (Figure 3C). Based on the results of Western blot analysis, the expression of CDH1 in the oe‐CDH1 group significantly increased compared with the oe‐NC group (*P* < .05) (Figure 3D). These results reveal that silencing and overexpression plasmids of CDH1 are successfully constructed.

### 3.3 Silencing of lncRNA H19, overexpression of CDH1, or demethylation of CDH1 inhibits sphere‐forming ability and invasion and promotes the apoptosis of A549 cells

**Figure 4:**
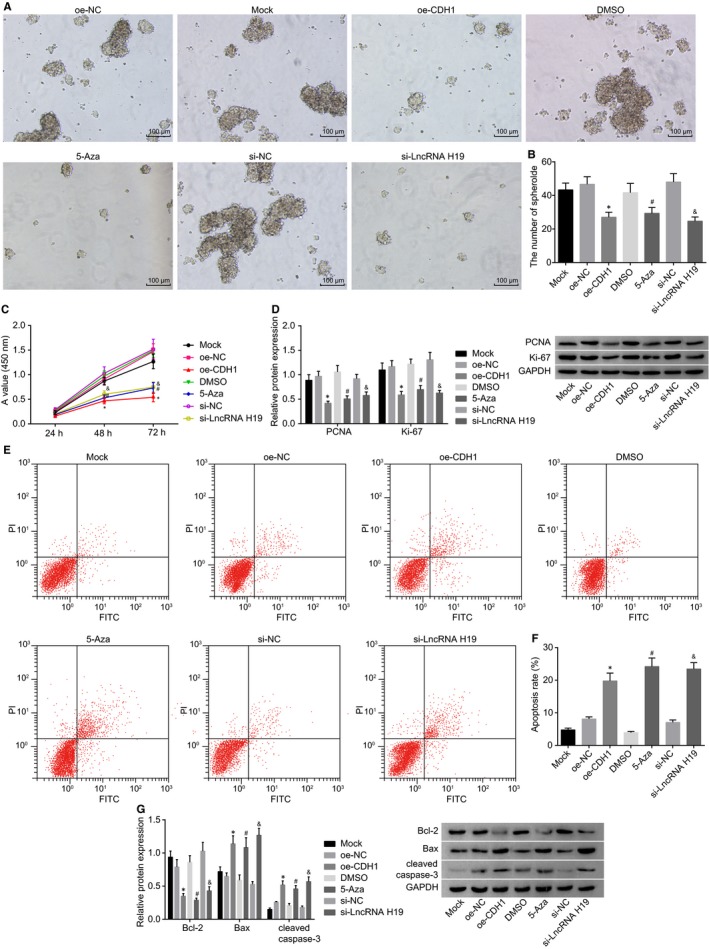
Sphere‐forming assay, CCK‐8 assay and flow cytometry demonstrate that silencing of lncRNA H19, overexpression of CDH1 or demethylation of CDH1 inhibits sphere‐forming ability and invasion of A549 cells and promotes the apoptosis. A and B, sphere‐forming ability of A549 cells in each group after transfection detected by sphere‐forming assay; C, cell proliferation of A549 cells measured by CCK‐8 assay; D, expression of PCNA and Ki‐67 determined by Western blot analysis; E, cell apoptosis measured by flow cytometry; F, statistical plot of A549 cell apoptosis in which the upper left quadrant identifies the necrotic cells (annexin V−/PI+), the upper right quadrant identifies the late apoptotic cells (annexin V+/PI+), the lower left quadrant identifies the live cells (annexin V‐/PI‐), and the lower right quadrant identifies the early apoptotic cells (annexin V+/PI‐); G, expression of Bax, Bcl‐2 and cleaved caspase‐3 determined by Western blot analysis. lncRNA H19, long non‐coding RNA H19; CDH1, E‐cadherin; CCK‐8, cell counting kit‐8; PI, propidium iodide; PCNA, proliferating cell nuclear antigen; Bcl‐2, B‐cell lymphoma/lewkmia‐2; Bax, Bcl‐2‐associated X protein; NC, negative control; DMSO, dimethylsulfoxide; GAPDH, glyceraldehyde‐3‐phosphate dehydrogenase. *P < .05 vs the oe‐NC group; ^#^ *P* < .05 vs the DMSO group; ^&^ *P* < .05 vs the si‐NC group. The measurement data are presented as mean ± standard deviation, analysed by one‐way analysis of variance. n = 3. The experiment was independently repeated three times

In order to investigate the effects of CDH1 and lncRNA H19 on the proliferation and apoptosis of lung adenocarcinoma cells, we transfected the previously constructed plasmids into A549 cells, followed by sphere‐forming assay, CCK‐8 assay and flow cytometry (Figure 4A,C,E,F). The results showed that there were no significant differences in sphere‐forming ability, proliferation or apoptosis of A549 cells among the mock, oe‐NC, si‐NC and oe‐CDH1 groups (*P* > .05). In comparison with the oe‐NC group, A549 cells in the oe‐CDH1 group exhibited significantly decreased sphere‐forming ability and proliferation, as well as significantly increased apoptosis (Figure 4B, *P* < .05). Compared with the DMSO group, the sphere‐forming ability and proliferation of A549 cells significantly decreased, and the apoptosis rate notably increased in the 5‐Aza group (*P* < .05). The si‐lncRNA H19 group showed a marked decrease in sphere‐forming ability and proliferation of A549 cells as well as a notable increase in apoptosis in comparison with the si‐NC group (*P* < .05). As shown in Western blot analysis (Figure 4D,G), compared with the oe‐NC group, the oe‐CDH1 group had significantly lower expressions of PCNA and Ki‐67, higher expressions of Bax and cleaved caspase‐3, and lower expression of Bcl‐2 in A549 cells (*P* < .05). Compared with the DMSO group, the expressions of PCNA and Ki‐67 significantly decreased, the expression of Bax and cleaved caspase‐3 increased, and the expression of Bcl‐2 decreased in A549 cells in the 5‐Aza group (*P* < .05). Compared with the si‐NC group, the expressions of PCNA and Ki‐67 significantly decreased, the expressions of Bax and cleaved caspase‐3 increased, and the expression of Bcl‐2 decreased in A549 cells in the si‐lncRNA H19 group (*P* < .05). Taken together, overexpression of CDH1, silencing of lncRNA H19 or the use of 5‐Aza could inhibit sphere‐forming ability and proliferation and promote the apoptosis of A549 cells, suggesting that the expression of lncRNA H19 or CDH1 is correlated with A549 cell proliferation.

### 3.4 Silencing of lncRNA H19, overexpression of CDH1 or inhibition of CDH1 methylation suppresses migration and invasion and EMT of A549 cells

**Figure 5:**
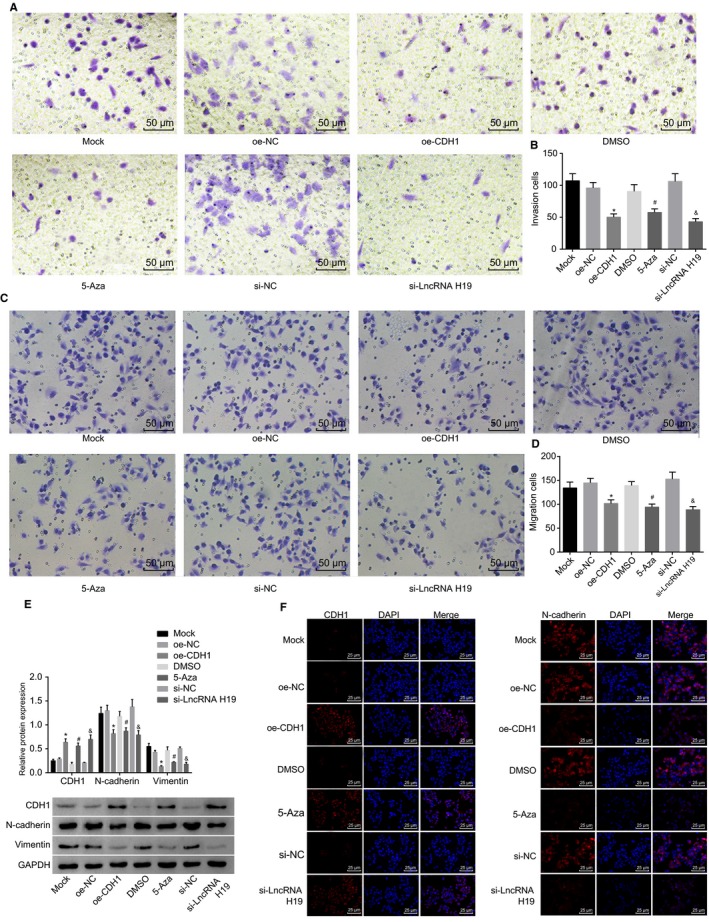
Scratch test and Transwell assay and immunofluorescence show that silencing of lncRNA H19, overexpression of CDH1 or inhibition of CDH1 methylation suppresses migration and invasion and EMT of A549 cells. A and B, migration ability of A549 cells in each group after transfection detected by scratch test; C and D, invasion ability of A549 cells in each group after transfection measured by Transwell assay; E, protein bands and statistical plot of expression of EMT‐related genes determined by Western blot analysis; F, expression of EMT‐related genes measured by immunofluorescence assay. lncRNA H19, long non‐coding RNA H19; CDH1, E‐cadherin; NC, negative control; DMSO, dimethylsulfoxide; EMT, epithelial‐mesenchymal transition; GAPDH, glyceraldehyde‐3‐phosphate dehydrogenase. *P < .05 vs the oe‐NC group; ^#^ *P* < .05 vs the DMSO group; ^&^ *P* < .05 vs the si‐NC group. The measurement data are presented as mean ± standard deviation, analysed by one‐way ANOVA. n = 20. The experiment was independently repeated three times

For investigation into the effects of CDH1 and lncRNA H19 on the EMT process, we further performed scratch test and Transwell assay to ascertain whether silencing of lncRNA H19, overexpression of CDH1 or inhibition of CDH1 methylation could affect migration and invasion of lung adenocarcinoma cells (Figure 5A,B,C,D). There were no significant differences in migration and invasion of A549 cells among the mock, oe‐NC, si‐NC and DMSO groups (*P* > .05). Compared with the oe‐NC group, the migration and invasion of A549 cells in the oe‐CDH1 group evidently decreased (*P* < .05). Compared with the DMSO group, the migration and invasion of A549 cells were significantly decreased in the 5‐Aza group (*P* < .05). The cell migration and invasion of A549 cells in the si‐lncRNA H19 group were significantly lower than those in the si‐NC group (*P* < .05). It was concluded that silencing of lncRNA H19, overexpression of CDH1 or inhibition of CDH1 methylation could suppress migration and invasion of lung adenocarcinoma cells. The effects of CDH1/lncRNA H19 on the EMT‐related factors in lung adenocarcinoma cell line A549 were explored by Western blot analysis. The results (Figure 5E) showed that there were no significant differences in the expressions of EMT‐related factors in the mock, oe‐NC, si‐NC and DMSO groups. Lower N‐cadherin and vimentin protein expressions as well as higher CDH1 protein expression were found in the oe‐CDH1 group in comparison with the oe‐NC group (*P* < .05). The 5‐Aza group exhibited significantly decreased N‐cadherin and vimentin protein expressions, in addition to increased CDH1 protein expression than the DMSO group (*P* < .05). In comparison with the si‐NC group, the si‐lncRNA H19 group showed markedly decreased N‐cadherin and vimentin protein expressions, in addition to increased CDH1 protein expression.

The effects of CDH1 and lncRNA H19 on EMT‐related factors in lung adenocarcinoma cell line A549 were further detected by immunofluorescence. The results (Figure 5F) showed that A549 cells in the mock, oe‐NC, DMSO and si‐NC groups had no CDH1 expression or had a poor expression, with the fields of vision presenting black colour and without any fluorescent staining; however, the N‐cadherin expression was high and a large amount of fluorescent staining was found in the fields of vision. In the oe‐CDH1, 5‐Aza and si‐lncRNA H19 groups, the A549 cells exhibited significantly decreased N‐cadherin expression, lesser or no fluorescent staining, significantly increased CDH1 protein expression, in addition to visible fluorescence in the fields of vision. The above results, which were in consistency with those of the Western blot assay, further demonstrated that silencing of lncRNA H19, overexpression of CDH1 or inhibition of CDH1 methylation by demethylating agent 5‐Aza could suppress EMT of A459 cells.

From the aforementioned results, silencing of lncRNA H19, overexpression of CDH1 or inhibition of CDH1 methylation could suppress migration and invasion and EMT of A549 cells.

### 3.5 Silencing of lncRNA H19 inhibits methylation of CDH1

**Figure 6:**
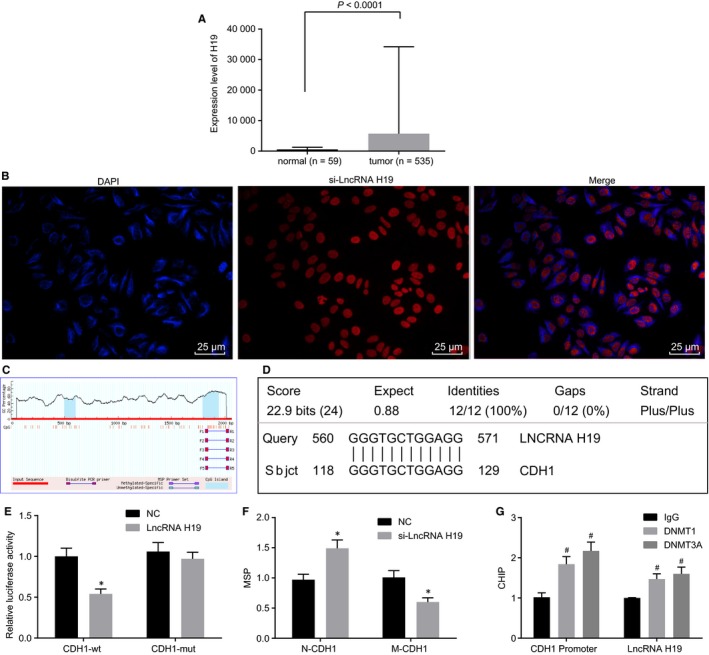
RNA‐FISH and CHIP reveal that silencing of lncRNA H19 inhibits methylation of CDH1. A, expression of H19 in lung adenocarcinoma tissues and normal tissues retrieved from the TCGA database; B, subcellular localization of lncRNA H19 detected by RNA‐FISH. C, CpG island in the CDH1 promoter region based on the Meth Primer software; D, the binding site between lncRNA H19 and CDH1 promoter predicted by biological website; E, the luciferase intensity of the CDH1 wild‐type promoter region detected by luciferase reporter gene; F, the luciferase intensity of the M‐CDH1 determined by luciferase reporter gene; G, statistical chart of CHIP. lncRNA H19, long non‐coding RNA H19; CDH1, E‐cadherin; NC, negative control; DMSO, dimethylsulfoxide; RNA‐FISH, RNA fluorescence in situ hybridization; ChIP, chromatin immunoprecipitation; M‐CDH1, methylated CDH1; DNMT1, DNA methyltransferase 1; DNMT3A, DNA methyltransferase 3A; GAPDH, glyceraldehyde‐3‐phosphate dehydrogenase. *P < .05 vs the NC group; ^#^ *P* < .05 vs the IgG group. The measurement data are presented as mean ± standard deviation, analysed by Student's *t* test. n = 3. The experiment was independently repeated three times

We downloaded lncRNA H19 expression in multiple cancers from the TCGA database and found that compared with normal tissues, lncRNA H19 was highly expressed in tissues of lung adenocarcinoma (Figure 6A). We designed probe primers of lncRNA H19, followed by FISH experiment (Figure 6B) to detect the subcellular location of lncRNA H19. The results showed that lncRNA H19 was located in the nucleus. After Merge, the nucleus had a fluorescent staining, suggesting that lncRNA H19 was highly expressed in the nucleus of the A549 cell line. Next, we input nucleotide sequences (3000 bp) near the CDH1 gene promoter in the Meth Primer software so as to analyse the CpG island in the CDH1 promoter region. The results showed that there was a CpG island in the CDH1 promoter region (Figure 6C). A biological prediction website was used to predict the target binding site of lncRNA H19, as shown in Figure 6D, and luciferase gene reporter was employed to verify whether CDH1 is a target gene of lncRNA H19. The results showed that in comparison with the NC group, the luciferase activity of the CDH1 wild‐type promoter region was significantly reduced in the si‐lncRNA H19 group (*P* < .05), while the luciferase activity of the mutant promoter region did not differ significantly (*P > *.05) (Figure 6E), indicating that lncRNA H19 specifically bind to the promoter region of CDH1. Subsequently, we continued to study the relationship between lncRNA H19 and methylation of CDH1 using double luciferase (Figure 6F), the results of which demonstrated that compared with the NC group, the expression of M‐CDH1 in the si‐lncRNA H19 was significantly reduced and the expression of N‐CDH1 increased.

For further verification, we performed ChIP assay (Figure 6G). After incubation with DNMT1 and DNMT3A antibodies, the results showed that the expression of lncRNA H19 and the expression of CDH1 promoter region in the DNMT1 and DNMT3A groups were significantly higher than those in the IgG group. LncRNA H19 induced DNMT1 and DNMT3A, which were enriched around the CDH1 promoter, to promote the methylation of CDH1.

Taken together, silencing of lncRNA H19 could inhibit methylation of CDH1.

### 3.6 Silencing of lncRNA H19, overexpression of CDH1 or inhibition of CDH1 methylation inhibits tumorigenicity of A549 cells in nude mice

**Figure 7:**
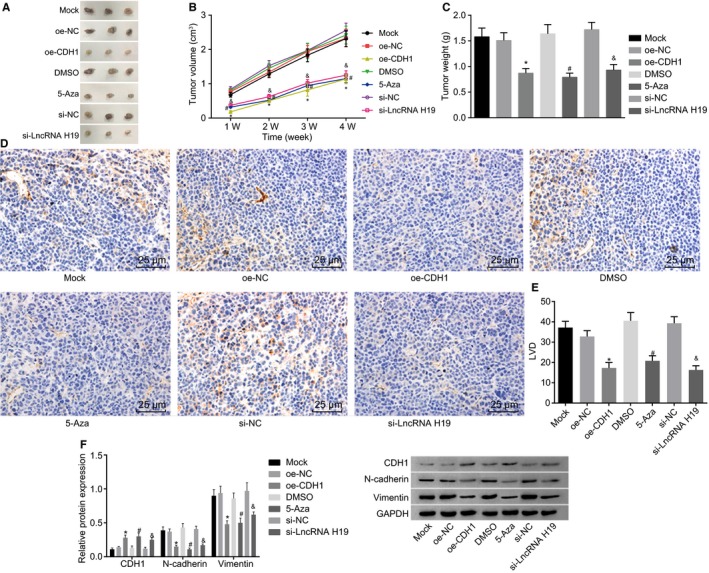
The results of xenograft in nude mice show that silencing of lncRNA H19, overexpression of CDH1, or inhibition of CDH1 methylation inhibits tumorigenicity of A549 cells in nude mice. A, B and C, images of tumour tissue morphology and statistical plots of tumour volume and tumour weight; D, positive expression rate of LYVE‐1 measured by immunohistochemistry; E, statistical chart of LVD; F, protein expression of EMT‐related genes determined by Western blot analysis. lncRNA H19, long non‐coding RNA H19; CDH1, E‐cadherin; NC, negative control; DMSO, dimethylsulfoxide; EMT, epithelial‐mesenchymal transition; LYVE‐1, lymphatic vessel endothelial hyaluronan receptor 1; LVD, lymphatic micro‐vessel density. *P < .05 vs the oe‐NC group; ^#^ *P* < .05 vs the DMSO group; ^&^ *P* < .05 vs the si‐NC group. The measurement data are presented as mean ± standard deviation, analysed by one‐way ANOVA. n = 6. The experiment was independently repeated three times

A nude mouse model of tumour formation was established. The tumour length and width were measured at the 1st, 2nd, 3rd and 4th week, respectively. The tumour tissues of nude mice were weighed at the 4th week. The results showed that there were no significant differences in volume and weight of the tumour tissues among the mock, oe‐NC, si‐NC and DMSO groups at the 1st, 2nd, 3rd and 4th week. Compared with the oe‐NC group, the volume and weight of tumour tissues in the oe‐CDH1 group decreased significantly (*P* < .05). Compared with the DMSO group, the 5‐Az group exhibited notably reduced volume and weight of tumour tissues (*P* < .05). Compared with the si‐NC group, a marked decrease in the volume and weight of tumour tissues was detected in the si‐lncRNA H19 group (*P* < .05) (Figure 7A,B,C). We further performed immunohistochemistry to determine the positive expression of the transfer specific factor LYVE‐1 (Figure 7D), and the average value of the 5 regions was used as the LVD of the group (Figure 7E). The positive expression of LYVE‐1 was found in each group under the microscope. No significant differences in positive expression of LYVE‐1 and LVD were detected among the mock, oe‐NC, si‐NC and DMSO groups. However, in comparison with the oe‐NC group, the positive expression of LYVE‐1 and LVD decreased significantly in the oe‐CDH1 group (*P* < .05). The positive expression of LYVE‐1 and LVD value were significantly lower in the 5‐Aza group than the DMSO group (*P* < .05). The si‐lncRNA H19 group exhibited a notably reduced positive expression of LYVE‐1 and a decreased valued of LVD in comparison with the si‐NC group.

The expressions of EMT‐related factors in the tumour tissues were determined by Western blot analysis (Figure 7F). The results exhibited no significant differences in the expressions of EMT‐related factors among the mock, oe‐NC, si‐NC and DMSO groups. Compared with the oe‐NC group, the expressions of N‐cadherin and vimentin significantly decreased, and the CDH1 protein expression significantly increased in the oe‐CDH1 group (*P* < .05). In comparison with the DMSO group, the 5‐Aza group exhibited significantly reduced expressions of N‐cadherin and vimentin as well as elevated CDH1 protein expression (*P* < .05). Compared with the si‐NC group, the expressions of N‐cadherin and vimentin significantly decreased in the si‐lncRNA H19 group, and the CDH1 protein expression increased (*P* < .05). The above results indicate that overexpression of CDH1, silencing of lncRNA H19 or use of demethylating agent can inhibit the tumour formation and metastasis of lung adenocarcinoma cells in nude mice.

## 4 DISCUSSION

Lung adenocarcinoma is a major contributor to cancer‐related death all over the world.^3^ LncRNAs are considered as biomarkers as well as molecular targets for cancer therapies.^18^ In this study, we investigated the role of lncRNA H19 and CDH1, methylation of CDH1 in lung adenocarcinoma and found that in contrary to CDH1, lncRNA H19 and methylation of CDH1 played a positive role in the pathological process of lung adenocarcinoma.

At first, we found that lncRNA H19 and methylation of CDH1 were highly expressed in lung adenocarcinoma tissues compared with the normal adjacent tissues, while CDH1 expression was reciprocal. LncRNAs are involved in the pathogenesis of multiple cancers, comprising lung adenocarcinoma.^19^ Consistently, Cheng et al^20^ demonstrated that lncRNA H19 was highly expressed in lung cancer cells. LncRNA H19 was found to participate in the occurrence of non–small‐cell lung cancer and to have a positive correlation with the tumour staging and poor prognosis.^21^ Similarly, Li et al^22^ also reported that lncRNA H19 promoted carcinogenesis as well as metastasis of gastric cancer. Methylation of DNA plays a critical role in cancer pathologies.^23^ Methylation of CDH1, which can induce the inhibition of CDH1 expression, is responsible for an elevated danger of lung cancer.^24^ In line with our study, a previous study conducted by Nakata et al reported that patients with non–small‐cell lung carcinoma who showed poorly expressed CDH1 and elevated level of CDH1 methylation suffered from a poorer prognosis in comparison with those who did not.^25^ Moreover, DNA methylation of CDH1 was found to be aberrantly expressed in rats with lung adenocarcinoma,^26^ which was consistent with the present study.

One of the key findings of our study was that silencing of lncRNA H19, overexpression of CDH1 or reducing CDH1 methylation suppressed proliferation, migration and invasion, promoted apoptosis of lung adenocarcinoma A549 cells and inhibited the EMT. EMT is a process primarily characterized by E‐cadherin loss and cell invasion and is regarded as a mechanism for cancer dissemination.^27^ Accumulating reports have demonstrated that lncRNAs are capable of affecting biological activities of cancer and participate in development of multiple diseases, via regulation of cell proliferation.^28^, ^29^ In addition, lncRNAs are identified as emerging regulators in metastatic cascade, among which lncRNA H19 has been found to be able to regulate EMT, thereby affecting tumour cell metastasis.^30^ Overexpression of lncRNA H19 is responsible for tumorigenesis and progression of diverse cancer types; lncRNA H19, serving as a microRNA sponge, could accelerate the process of EMT in colorectal cancer.^31^ Moreover, lncRNA H19 stimulated invasion and migration of let‐7 in pancreatic ductal adenocarcinoma, in part through the up‐regulation of HMGA2‐mediated EMT.^32^ The above reports all supported our results that overexpressed lncRNA H19 could accelerate the development of lung adenocarcinoma cells, while by silencing of lncRNA H19, this trend could be significantly reversed. CDH1 is considered as an anti‐oncogene in interactions between epithelial cells.^33^ Reduced CDH1 expression is referred to as a biomarker for cancer invasion as well as metastasis.^34^ DNA methylation is one of the epigenetic changes that account for aberrant regulation of tumour‐related genes, which can affect cellular activities such as cell proliferation, apoptosis and metastasis.^35^ Consistent with our results, inhibition of CDH1, identified as a critical gene during EMT process, was found to aid in tumour metastasis of breast cancer.^36^ Therefore, it has been suggested that lncRNA H19 might affect cellular processes of lung adenocarcinoma cells by regulating methylation of CDH1.

Furthermore, we found that silencing of lncRNA H19 could inhibit methylation of CDH1 promoter in lung adenocarcinoma. Accumulating evidence demonstrates that lncRNAs are able to mediate transcription as well as DNA methylation of CpG island.^37^ LncRNA H19 has been revealed to be capable of interacting with histone lysine methyltransferases.^38^ Moreover, lncRNA H19 could bind to S‐adenosylhomocysteine hydrolase (SAHH) and repressed its expression, thereby leading to DNMT3B, which produced methylate gene subsets.^39^ It was reported that silencing of lncRNA H19 could elevate Nctc1 methylation which was mediated by DNMT3B.^40^ Additionally, lncRNA H19 was found to be able to regulate expression of Hnf4α as well as its methylation.^41^ In the current study, the ChIP assay showed that lncRNA H19 induced DNMT1 and DNMT3A to promote the methylation of CDH1; however, the potential mechanism implicated in link of LncRNA H19 with methyltransferases in lung adenocarcinoma cells still remained complicated.

**Figure 8:**
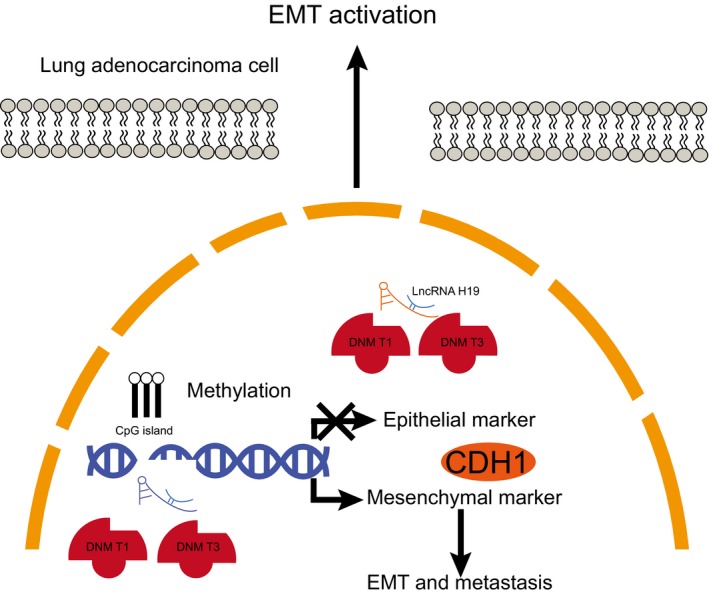
Molecular mechanisms of lncRNA H19 involved in lung adenocarcinoma by promoting methylation of CDH1 promoter. LncRNA H19 is highly expressed in lung adenocarcinoma. LncRNA H19 recruits methyltransferases DNMT1 and DNMT3A to CDH1 promoter region, which leads to up‐regulation of the expression of CDH1D, thereby promoting proliferation and EMT while inhibiting apoptosis of lung adenocarcinoma cells. lncRNA H19, long non‐coding RNA H19; CDH1, E‐cadherin; DNMT1, DNA methyltransferase 1; DNMT3A, DNA methyltransferase 3A; EMT, epithelial‐mesenchymal transition

To conclude, the present study demonstrated that silencing of lncRNA H19 inhibited EMT and proliferation while promoting apoptosis of lung adenocarcinoma cells via inhibition of methylation of CDH1 promoter (Figure 8), which may provide a novel molecular target for treatment of lung adenocarcinoma. However, the specific mechanism by which lncRNA H19 inhibits methylation of CDH1 promoter still lacks elucidation. Therefore, further study is required for validating our findings. In addition, we plan to supplement BSP experiment and to explore the relationship between lncRNA H19 and DNMT3B in the later experiment if possible.

## CONFLICTS OF INTEREST

The authors have declared no conflicts of interest.

## **AUTHOR** **CONTRIBUTIONS**

LMG, SFX, YZ, YL, JZ, QT and XBY designed the study; YZ, YL, JZ, XBY and LZ collected the data; LMG, SFX, PW, LZ, SSS and TW analysed the data; LMG, SSS, TW and YL interpreted the data; SFX, YZ, PW, LZ and QT drafted the manuscript; and LMG, SFX, YZ, PW, LZ, SSS, TW, YL, JZ, QT, XBY and LZ approved the final version of the manuscript.

## Data Availability

The data used to support the findings of this study are available from the corresponding author upon request.
